# Point-of-Need Additive Manufacturing in Austere Arctic Environments: An Evaluation of Medical Logistics Requirements and Capabilities Demonstration

**DOI:** 10.3390/bioengineering11030232

**Published:** 2024-02-28

**Authors:** Cate Wisdom, Nicholas Chartrain, Kelli Blaize-Wise, George J. Klarmann, Kristin H. Gilchrist, Vincent B. Ho

**Affiliations:** 1 4DBio^3^ Center for Biotechnology, Uniformed Services University of the Health Sciences, 9410 Key West Ave., Suite 150, Rockville, MD 20850, USA; emily.wisdom.ctr@usuhs.edu (C.W.); thewiseoptionc@gmail.com (K.B.-W.);; 2 The Geneva Foundation, 950 Broadway, Suite 307, Tacoma, WA 98402, USA; 3 Department of Radiology and Radiological Sciences, Uniformed Services University of the Health Sciences, 4301 Jones Bridge Road, Bethesda, MD 20814, USA

**Keywords:** 3D printing, additive manufacturing, point-of-need manufacturing, austere operations, medical logistics, bioprinting

## Abstract

Medical response to military conflicts, natural disasters, and humanitarian crises are challenged by operational logistics with unreliable supply chains, delayed medical evacuation, and compatibility of the disparate medical equipment and consumables. In these environments, stocks of supplies will become more quickly depleted and the need for equipment parts increases secondary to their higher likelihood for failure from overuse. Additive Manufacturing (AM), or 3D printing, at or closer to the point-of-need provides potential solutions to mitigate these logistics challenges. AM’s ability to tailor the resultant product through computer design enables real-time modification of a product to meet a specific situation. In this study, we deployed two different 3D printers to an arctic locale to demonstrate the utility of 3D printing and bioprinting in austere environments. Deployment of AM solutions in austere environments will likely impact medical care following natural disasters and conflicts with contested logistics. The work presented here furthers the readiness status of AM for use in austere environments to manufacture medical equipment parts and demonstrates its potential use for tissue engineering and advanced medical treatments in remote environments.

## 1. Introduction

Operational military medical facilities face logistics and resupply challenges not typical to garrison military medical facilities or civilian hospitals. Challenges inherent to operational medical facilities include risk of adversaries attacking the facilities or resupply deliveries, difficult access without infrastructure supporting normal ground delivery vehicles, and the unpredictable nature of war zones [1]. Military experts have predicted that care for our wounded will depend more heavily on prehospital field care for prolonged periods [2]. Mass casualty events further diminish critical medical material during periods where resupply is difficult or impossible. Stocks of consumable medical supplies may become depleted, and broken medical equipment may not be able to be repaired or replaced. Prolonged medical care in these scenarios may substantially raise mortality and morbidity rates. Furthermore, military facilities in operational environments must be prepared to operate without resupply for indeterminate periods of time.

In addition to military medical environments, natural disasters, and other humanitarian crises often produce similar challenges with availability of medical material, requirements for prolonged field care, and quickly diminishing supplies. The United Nations Global Humanitarian Overview indicated that 274 million people were in need of assistance during 2021 [3]. Logistics for humanitarian efforts are complex, and it is difficult to forecast demand due to the unpredictable nature of disasters and reliance on donations [4]. Medical materials received from disparate organizations, nations, and readily available on the market frequently do not integrate to enable appropriate safety and function. Like multi-national military efforts and the unpredictable nature of conflicts, a unique solution is required to enable access to medical equipment and replacement parts to facilitate care for people affected by disasters and humanitarian crisis.

Additive Manufacturing (AM) (or 3D printing) offers a solution to some of the logistical challenges of prolonged care in austere environments. AM is a broad term that encompasses many different types of 3D printing technologies, including selective laser sintering (SLS), stereolithography (SLA), inkjet 3D printing, fused deposition modeling (FDM), extrusion printing, and laser-assisted bioprinting are all forms of 3D printing [5]. 3D printing can be used to fabricate three dimensional parts from a wide variety of materials (e.g., plastics, metals, ceramics) based on a digital model, also called a computer-aided design (CAD) file. AM at the point-of-need allows for production of objects such as replacement parts and tools on-site, that could mitigate reliance on traditional supply chains, with clear benefits to austere medical facilities deployed in remote locations. SLA, FDM, and extrusion printing are good choices for AM in austere locations due to their simplicity and reliability. The relative simplicity of the three-axis gantry underpinning many AM technologies, including FDM and extrusion bioprinting provides sufficient reliability for transportation to and use in austere locations. The utility of AM for the manufacture of personal protective equipment and medical device parts was exemplified during supply chain disruptions at the outset of the COVID-19 pandemic [6]. Furthermore, in the medical field, AM is used to make prosthetics, orthopedic implants, dental repair implants, and medical tools [5,7,8].

3D bioprinting, or extruding materials with biological components, is being investigated for a range of advanced treatments including manufacturing of engineered tissues and bioactive therapeutics. While the use of 3D printing for engineered tissues is the subject of considerable research, significant additional advanced developments are needed before it can be implemented for patient treatment [9,10,11]. It is important to stress that all 3D printed medical devices or biologicals/cellular therapies intended for use in patient treatments need to follow regulatory compliance for use in human subjects and adhere to governmental guidance for quality and safety standards of the host nation’s regulatory agency, such as the FDA [12]. As these treatments advance to clinical use, the application of such biotechnologies may be found to be clinically most advantageous if applied early even if in austere, far-forward locations. Admittedly, these biologic technologies will require more sophisticated medical support, as supplies are often perishable and their transport to remote or hostile environments is problematic. 3D bioprinting, nonetheless, is becoming a more viable medical solution that has the potential to improve patient outcomes and help support extended care in austere locations.

As shown during the COVID 19 pandemic, PPE and equipment parts were in high demand and low supply secondary to manufacturing shutdowns and disrupted supply chains, especially for products manufactured overseas. During the pandemic, 3D printing enthusiasts and laboratories focused efforts to meet their local needs using rapid 3D production to replenish quickly diminishing stocks of critically needed PPE [13]. In addition to helping to supply PPE, 3D printing was utilized to provide lifesaving ventilator valves, although methods to safely incorporate them into the patient care environment had to be carefully considered [14]. While the manufacture of medical devices and replacement parts is best carried out in regulated facilities or medical centers, in times of dire need, including military conflicts, pandemics, and natural disasters, AM could be leveraged to improve healthcare and lives. In such scenarios, 3D printing could maximize operational availability of lifesaving equipment by manufacturing replacement parts at the point-of-need. The FDA and other regulatory agencies may choose to relax requirements during declared emergencies, such as during the COVID-19 pandemic, through the issuance of an emergency use authorization (also known as an EUA), as long as 3D printed or improvised devices create no undue risk, as noted for face masks and barrier face coverings [13,15,16].

3D printers can be integrated into Role 2 and Role 3 medical facilities to support the manufacture of replacement parts and consumables that become quickly depleted and bring advanced bioprinting therapies to austere environments. Role 2 medical support, typically at the Brigade level, includes triage, resuscitation, treatment, and holding of patients, whereas Role 3 support, typically at the Division level, includes additional surgical and specialist capabilities [17]. Filament-based 3D printers could be integrated into Role 2 and 3 facilities. Due to the requirements for sterility and surgical capabilities for many bioprinting technologies, most applications for bioprinting would apply to Role 3 or higher levels of care. Both Role 2 and 3 facilities have the capability to generate power and support 3D printers. Additionally, custom 3D printers can be designed to operate from vehicle or equipment batteries [18]. While raw materials in the form of thermoplastic filament spools or shelf stable biomaterials would need to be pre-positioned and replenished, a wide array of parts can be manufactured from one spool of material, decreasing storage space requirements, and the number and types of parts that must be replenished. The US Department of Defense (DoD) vendor agreements require shipment of replacement parts within 7 days of ordering for predicted items and volume. If volume requirements exceed what is anticipated, like in a mass casualty event, the vendor has up to 30 days to ship replacement items. For items that have not been ordered in the last 30 days, like broken equipment parts, the vendor has up to 90 days to ship replacement items [19].

In this study, we deployed a commercially available plastic filament 3D printer, and a military specific ruggedized filament and bioprinter to a Role 3 facility in an arctic locale to demonstrate the wide-ranging utility of AM in austere environments. Additionally, we demonstrated the ability to deploy 3D printers and supplies to prepositioned locations using a commercial shipping carrier and commercial passenger aircraft.

## 2. Materials and Methods

### 2.1. Commercial Thermoplastic Filament Printer

A standard commercial filament-based 3D printer with air handler (Ultimaker S5, Ultimaker, Utrecht, The Netherlands) was used to manufacture parts from spools of 2.85 mm diameter thermoplastic filament. A filament dryer (PrintDry PRO, PrintDry, Windsor, ON, Canada) removed moisture from filaments to ensure printability and consistency in a humid environment. A voltage inverter was used to run the filament dryer on 220V European outlets. Commercial filament materials were purchased and transported to the test site. Materials included Tough PLA (polylactic acid), PC (polycarbonate), PA6-CF (nylon carbon fiber), TPU-95A (polyurethane), and PVA (polyvinyl alcohol) support material. Materials were printed using temperatures recommended by the manufacturer. CAD files of medical materials and consumables were sourced online (Thingiverse) or constructed using CAD software (Autodesk Inventor 2023 and Autodesk Netfabb Premium 2020). CAD files were converted into .stl file format and uploaded into slicing software (Ultimaker Cura Enterprise 1.10.0). Material and print parameters were selected in the slicing software, and the print file was saved to a USB drive as an .ufp (Ultimaker Format Package) file and uploaded to the commercial printer. Parts with protrusions or overhangs were printed using the PVA support material to enable printing of parts with large overhangs or complex geometries. These prints were soaked in water after completion to remove the support material.

### 2.2. Ruggedized 3D Printer and Bioprinter

A custom ruggedized 3D printer (nRugged, nScrypt, Orlando, FL, USA) was used to print tissue-engineered meniscus constructs and thermoplastic parts. The .stl files were exported from CAD software and uploaded into MTGen 3.7.6 software (nScrypt, Orlando, FL, USA) for slicing and optimizing print parameters. MTGen software was used to operate the ruggedized printer. The tissue-engineered meniscus construct was printed at a speed of 20 mm/s and pressure of 8 kPa from an alginate and cellulose bioink (CELLINK Bioink, CellInk, Boston, MA, USA). The meniscus was crosslinked using a solution provided with the bioink. The thermoplastic parts were printed using spools of 1.75 mm diameter PLA (polylactic acid) thermoplastic filament.

### 2.3. 3D Scanner and Supporting Equipment

A 3D scanner (Einscan SP, Shining 3D, San Leandro, CA, USA) was used to generate unique CAD files from existing parts when digital models were not available. Digital scans were then post-processed (e.g., model smoothing, hole filling) and exported into a 3D printable format using ExScan S 3.1.2.0 software on a standard laptop computer. The scanner could be operated on a desktop by mounting the scanner and placing the part on a rotating plate. A snow mobile knob was scanned using the desktop configuration, and a CAD file was generated for printing.

### 2.4. Logistics, Shipping and Packaging

The commercial printer and associated support equipment were transported to the arctic locale via a commercial air carrier (United Airlines) as checked luggage. The commercial printer was packaged in the manufacturer-supplied cardboard box with foam inserts. The box was further reinforced and weatherproofed by placing it inside a canvas bag with wheels, which was secured using ratchet straps around the bag. The support equipment for the commercial printer, including the air handler, 3D scanner, and extra glass print beds, were packaged in a Pelican 0370 case. Two additional suitcases were used to pack the filament dryer, filament, print heads, tools, plug adapters, and a power inverter.

The ruggedized 3D printer consists of a road case used to package the printer, a Pelican 1730 case that contains the computer to run the printer, a compressor, and battery power inverter. The road case and Pelican case were packaged in a wooden pallet case (ULINE, Pleasant Prairie, WI, USA) and shipped via a commercial carrier (DHL). A second wooden pallet was shipped with a Pelican case containing a computer monitor and additional equipment. Equipment inside the pallet cases was secured with plastic moving wrap, and the void space was filled with cut construction grade foam board (FOAMULAR, Owens Corning, Toledo, OH, USA).

Both printers and all materials were packaged within the two wooden pallet crates and shipped back to the United States with the commercial carrier after the exercise concluded.

The commercial carrier was used to ship ready-to-print human mesenchymal stem cells (Rooster Bio). Two cryovials of 1 × 10^6^ cells were inserted into cryovial straws and inserted into a transportation grade liquid nitrogen dewar. The dewar was inserted into a secondary plastic container and secured with zip-ties.

## 3. Results and Discussion

### 3.1. Point-of-Need Manufacture of Replacement Parts with the Commercial Printer

**Figure 1:**
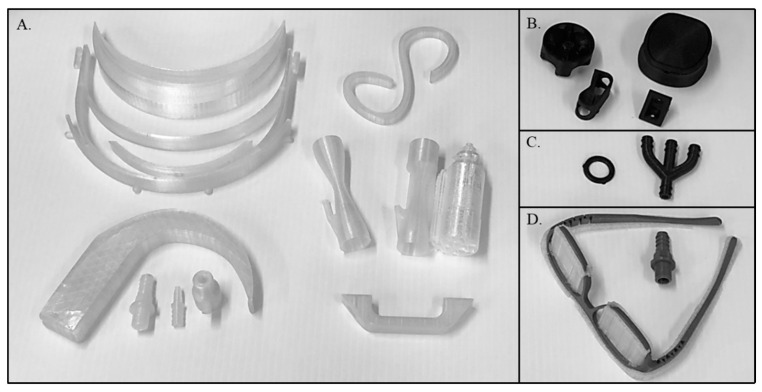
3D printed parts produced in the austere arctic locale. Parts were printed from Polycarbonate (**A**), Nylon Carbon Fiber (**B**), Polyurethane (**C**), and Tough PLA (**D**). A list of parts, print parameters, and cost per part are shown in Table 1. The set of three Polycarbonate ventilator valves are shown after the PVA support material was removed by washing with water (left and center) and with the support material intact (right). The clear material in the Tough PLA eye glass frames is PVA support material which would be removed and replaced with eye glass lenses.

The commercial printer successfully 3D printed a wide variety of medical equipment and repair parts (Figure 1). These included tubing connectors, ventilator connectors and valves, a laryngoscope, IV tubing clamps and holders, brackets, gaskets, handles, face shield parts, and glasses frames. Table 1 contains a summary of the materials, printing conditions, parts printed, material cost per part, and the print time per part.

Ventilator valves, which connect the ventilator to the patient’s airway support and control the flow of air and oxygen, are essential for intubation which is vital for treating respiratory failure or use during surgery. Ventilator valves are often transported with a critical care patient, leaving the austere healthcare facility with a shortage of valves for future patients requiring ventilation. In the same way, IV tubing clamps, tubing connectors, and gaskets, which may not necessarily be considered as consumable items, can be evacuated alongside casualties. The removal of such items will lead to reduced readiness of the healthcare facility to provide adequate support for future casualties.

Hose and tubing adaptors are essential for connecting different types of medical tubing and equipment. Although efforts have been made to standardize dimensions and connector types, differences between manufacturers can lead to incompatibilities. This issue is further exacerbated by the procurement of equipment from different vendors in the U.S. Air Force, Navy, and Army military medical systems, but particularly true for disparate medical equipment common to multi-national medical operations such as under the auspices of the Committee of the Chiefs of Military Medical Services in the North Atlantic Treaty Organization (NATO). Evacuation of patients from theatre may necessitate the transfer of patient care to a different Service or medical team from a different country. If that receiving party does not have the same life-sustaining equipment as the initial medical providers, the patient may require redundant equipment due to incompatibilities. In addition, differences in connector types between allied countries limit integration of medical equipment between coalition forces. Therefore, the ability to 3D print multipurpose connectors to interface between incompatible and disparate equipment would be of great importance in austere environments with contested logistics.

Replacing eyeglasses can be impractical, and intact frames are likely to provide more comfort to the wearer than a broken pair that have been taped or glued back together. The 3D printed eye glass frame is advantageous as it does not have a hinge, eliminating a common failure mode. The frames can be digitally resized to meet specific requirements of the Service member and integration of existing equipment. This is one example of how 3D printing can be used to fabricate replacement and individual customizable parts.

An additional intravenous (IV) bag holder can be useful when all available slots on a pole are taken, or when there is a mismatch between equipment and supplies, or environmental constraints to the care of the patient. The ability to digitally modify parts allows for the integration of common supplies into new systems and/or new environments. Custom IV bag holders can be designed to allow for hanging IV bags in certain vehicle or triage scenarios. While this is a simple example, in a resource constrained/mass casualty environment medical providers are responsible for significant numbers of patients, and hands-free administration of IV fluid or medication allows for providers to be available to treat additional patients.

Another advantage of 3D printing is that multiple parts can be fabricated simultaneously as need requires by arranging multiple copies of a part on the print bed in the slicing software. Printing multiple parts in same print process can result in slightly lower print times per part due to efficiencies that can be gained in the path that the printhead follows, especially when support material must be printed.

### 3.2. Point-of-Need Manufacture of an Equipment Essential Replacement Part

**Figure 2:**
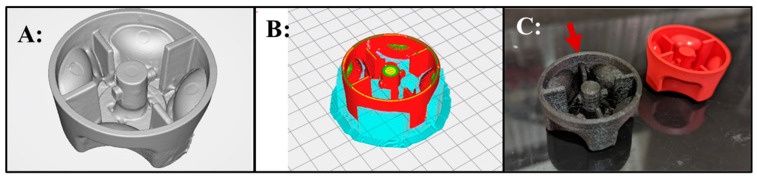
Demonstration of point of need capabilities. (**A**): 3D scanned and digitally repaired snowmobile knob that frequently needs replacement. (**B**): The repaired knob with PVA support material (blue) in the commercial printer slicing software. The grey color with grid lines in the background shows the location of the part on the 3D printer build platform indicated in the slicing software. (**C**): 3D printed snowmobile knob replacement part (arrow) and an unbroken knob.

During the exercise, the utility of point-of-need manufacturing was highlighted by an unplanned use-case scenario. A local military officer inquired if a broken snowmobile starter knob could be printed. These knobs frequently break, and the snowmobiles will not operate without the knob. Further, snowmobiles are an important transportation element for arctic operations. The snowmobile knob was 3D scanned, a CAD model created, the damaged section digitally repaired, and a functional new part 3D printed out of a carbon fiber filled nylon material (Figure 2). The complex interior of the knob required multiple 3D scans to be taken and meshed together to accurately capture the overhangs of the switch mechanism. The process of scanning and repair took approximately 2 h. The material cost of the 3D printed knob was approximately $3.28, including water dissolvable PVA support material. Normally, to replace a broken knob, an entire subsystem must be purchased from the manufacturer at an approximate cost of $900 USD. This simple demonstration made clear the benefits for an austere 3D printing capability for military forces, and would be even more pronounced in a contested logistics scenario during which replacements cannot be obtained from the manufacturer in a timely manner.

### 3.3. Point-of-Need Bioprinting with the Ruggedized Printer

**Figure 3:**
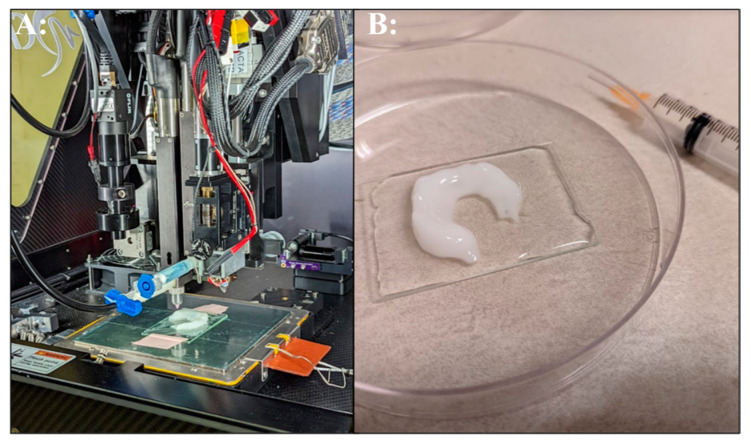
3D bioprinted meniscus. (**A**): Bioprinting alginate bioink on the ruggedized printer. (**B**): Completed bioprinted meniscus in post-processing crosslinking solution.

The ruggedized 3D printer successfully fabricated several parts and demonstrated the potential of bioprinting for tissue engineering in deployed locations. A knee meniscal model was derived from human anatomic scan data obtained from a manufacture’s software training files (Mimics Innovation Suite, Materialise, Plymouth, MI, USA). The meniscus model was chosen to evaluate the feasibility of bioprinting in an austere arctic military medical facility and to replicate our prior work conducted in a desert environment [18]. The meniscus was printed using a commercially available bioink (Figure 3). These results demonstrate the feasibility of extrusion 3D printing of hydrogels in austere environments, which, subject to FDA guidance, could someday be implemented for patient care in remote locations. In the prior desert environment study, human mesenchymal stem cells (MSC) were incorporated into the scaffold to more closely replicate tissue engineering approaches conducted in a research laboratory. However, in the current work, cells transported in a liquid nitrogen dewar were unable to clear customs in the host country and were shipped back to the United States, which prevented the evaluation of cellular bioink printing in this exercise. Material availability, cold chain requirements, and sterility are parameters that increase the challenge of implementing tissue engineering approaches in austere environments. In Role 3 facilities, cold storage equipment is in place. However, commercial shipping of cellular materials will be challenging and require authorization from host nation customs. One possible solution is to include cellular and other biological materials in military air shipments. Further research into lyophilization strategies for cellular therapeutics and methods to fabricate cell free tissue scaffolds could be most promising for manufacturing advanced scaffolds and therapies for deployment in austere environments.

### 3.4. Logistics Schemes

Two logistics schemes were evaluated. The first was the feasibility of rapidly deploying a 3D printing solution via a commercial air passenger carrier. For this evaluation, the commercial printer, scanner, filament dryer, tools, and materials were transported as checked luggage by two passengers. The second logistics scheme evaluated was the palletized shipment of the ruggedized printer and additional equipment by a commercial carrier. Table 2 depicts the items and logistics schemes for deploying the commercial and ruggedized printers to a rural arctic locale.

Shipping of the two 3D printers in two different manners mitigated risks in shipping logistics due to the short timeline prior to deployment. The team was given approximately thirty days of notice before personnel were due to depart for the austere arctic test site. In this short period, equipment was tested and validated to be in functional condition, items were packed, various shipping options evaluated, and the ruggedized 3D printer shipped via commercial air freight. Deployment of the printing assets through military logistics channels was not a viable option due, in part, to the short timeline.

Both the commercial and ruggedized printers and equipment arrived undamaged at the testing site due to ruggedization and careful packaging. Commercial shipping and customs clearance are a bottleneck for rapid deployment of equipment outside of the United States. The shipment of the wooden pallet cases to the rural arctic locale with clearance through host country customs took 14 days. The return shipment to the United States and clearance through US customs took 34 days. The time spent in host country customs was 7, days and the time spent in US customs was 26 days. The logistics timeline for the wooden pallet crates dispatch to the rural arctic locale and return to Rockville, MD, USA, is depicted in Table 3. Military logistics transportation lines could improve availability of the equipment, but for a smaller footprint solution, such as the commercial printer, rapid deployment by commercial passenger carriers is possible. To ensure timely availability, a better solution could be to have the entire ruggedized and commercial 3D printing solution pre-positioned with existing Role 2 or Role 3 medical infrastructure.

## 4. Conclusions

3D printing of medical equipment parts and consumables has the potential to support more effective medical care in Role 1–3 environments, although printers would likely be physically located at Role 2 or 3 echelons of care. Deployment of AM solutions in austere environments would likely have enormous impacts on medical care during conflicts that lead to contested logistics. The inability to repair essential medical equipment, obtain basic medical supplies, or provide novel solutions that may not be apparent (e.g., disparate equipment in multi-national medical operations) in such situations likely will result in delays in patient care and/or patient transfer and higher morbidity and mortality rates. The use of bioprinters for printing tissue scaffolds and future approved advanced therapies in austere environments has the potential to further support AM use in support of forward medical care. 3D printing was widely used at the outset of the COVID-19 pandemic as a rapid and distributed way of manufacturing personal protective equipment and medical device parts. Although minimal work has been demonstrated to employ AM in austere environments, its benefits during supply chain disruptions are clear. This work furthers the readiness status of AM for use in austere environments to manufacture medical equipment parts and demonstrates its potential use for tissue engineering and advanced medical treatments in deployed situations.

## Figures and Tables

**Table 1:** Summary of materials, material costs, print conditions, and parts printed to demonstrate point of need manufacturing in austere environments. The part cost and time were determined in using slicing software (Ultimaker Cura).

Material	Part Description	Part Cost (USD)	Print Time (hh:mm)
Polycarbonate (PC)	Face shield parts	$8.84	18:22
Nozzle/Bed: 280 °C/110 °C	Laryngoscope	$5.11	6:45
	Eye glass frames (not pictured)	$4.38	8:03
	Barbed tubing adaptor	$0.49	0:55
	Hose tubing adaptor	$0.15	0:23
	Ventilator tubing adaptor	$0.59	1:09
	IV bag holder	$1.43	2:42
	Ventilator valves (set of 3)	$9.31	16:56
	Handle	$1.16	1:43
Nylon Carbon Fiber (PA6-CF)	Snowmobile switch	$3.23	3:48
Nozzle/Bed: 265 °C/70 °C	Medical device housing	$9.27	9:17
	IV tubing clamp	$0.66	1:36
	Bracket	$0.31	0:45
Polyurethane (TPU-95A)	Gasket	$ 0.10	0:11
Nozzle/Bed: 223 °C/60 °C	Tubing connector	$0.62	1:33
Tough PLA	Eye glass frames	$3.81	7:41
	Barbed tubing adaptor	$0.32	0:47

**Table 2:** Details of equipment, transportation via commercial flight (grey rows) and commercial shipping carrier (white rows) from Washington DC to the rural arctic locale. All items were returned to the United States within the two wooden pallet cases.

	Items	Packaging	Weight	Dimensions	Required Fees
Scheme 1	Commercial 3D Printer	Manufacturer packaging, canvas zippered bag with wheels	67 lbs.	22″ × 25″ × 32″	Overweight, oversize
	3D Scanner, 3D Printer Airhandler, extra glass print beds	Pelican 0370 Case	65 lbs.	26.5″ × 26.5″ × 25.3″	Overweight, oversize
	Filament dryer, filament spools, print heads, tools, US to EU plug adapters, power inverter	2 large suitcases	65 lbs. each	29″ × 19″ × 10.5″	Overweight, extra bag
Scheme 2	Ruggedized 3D printer in Road Case with Casters	Pallet case	625 lbs.	48″ × 40″ × 42″	
	Accessories, peripherals, and materials for ruggedized 3D printer	Pallet case	250 lbs	48″ × 40″ × 42″	

**Table 3:** Shipment timeline of palletized equipment by a commercial carrier dispatched from a laboratory in Rockville, MD, to the rural arctic test site and the return shipment back to Rockville, MD.

Dispatch		
Day 0	Depart Laboratory	Rockville, MD, USA
Day 6	Host Country Customs	Host Country
Day 14	Delivery to Test Site	Rural Arctic Locale
Return		
Day 0	Depart Test Site	Rural Arctic Locale
Day 7	US Customs	Cincinnati, OH, USA
Day 34	Return to Laboratory	Rockville, MD, USA

## Data Availability

Dataset available on request from the authors.
